# Chronic cerebral hypoperfusion shifts the equilibrium of amyloid β oligomers to aggregation-prone species with higher molecular weight

**DOI:** 10.1038/s41598-019-39494-7

**Published:** 2019-02-26

**Authors:** Taro Bannai, Tatsuo Mano, Xigui Chen, Gaku Ohtomo, Ryo Ohtomo, Takeyuki Tsuchida, Kagari Koshi-Mano, Tadafumi Hashimoto, Hitoshi Okazawa, Takeshi Iwatsubo, Shoji Tsuji, Tatsushi Toda, Atsushi Iwata

**Affiliations:** 1 Department of Neurology, Graduate School of Medicine, The University of Tokyo, 7-3-1 Hongo, Bunkyo-ku, Tokyo, 113-8655 Japan; 2 Department of Neuropathology, Graduate School of Medicine, The University of Tokyo, 7-3-1 Hongo, Bunkyo-ku, Tokyo, 113-8655 Japan; 3 Department of Neuropathology, Graduate School of Medicine, Tokyo Medical and Dental University, 1-5-45 Yushima, Bunkyo-ku, Tokyo, 113-8510 Japan

## Abstract

Epidemiological studies have shown that atherosclerotic risk factors accelerate the pathological process underlying Alzheimer’s disease (AD) via chronic cerebral hypoperfusion. In this study, we aimed to clarify the mechanisms by which cerebral hypoperfusion may exacerbate AD pathology. We applied bilateral common carotid artery stenosis (BCAS) to a mice model of AD and evaluated how the equilibrium of amyloid β oligomers respond to hypoperfusion. BCAS accelerated amyloid β (Aβ) convergence to the aggregation seed, facilitating the growth of Aβ plaques, but without changing the total Aβ amount in the brain. Furthermore, Aβ oligomers with high molecular weight increased in the brain of BCAS-operated mice. Considering Aβ is in an equilibrium among monomeric, oligomeric, and aggregation forms, our data suggest that cerebral hypoperfusion after BCAS shifted this equilibrium to a state where a greater number of Aβ molecules participate in Aβ assemblies to form aggregation-prone Aβ oligomers with high molecular weight. The reduced blood flow in the cerebral arteries due to BCAS attenuated the dynamics of the interstitial fluid leading to congestion, which may have facilitated Aβ aggregation. We suggest that cerebral hypoperfusion may accelerate AD by enhancing the tendency of Aβ to become aggregation-prone.

## Introduction

Alzheimer’s disease (AD) is a chronic progressive neurodegenerative disorder, characterized by cognitive decline, including memory disturbance, and loss of executive function. AD is pathologically characterized by interstitial deposition of amyloid β (Aβ) and subsequent neuronal accumulation of phosphorylated tau, which ultimately lead to neuronal dysfunction and eventual neuronal death^1^. Aging is one of the strongest risk factors for the disease. Genetic factors are known to contribute as well, with the *APOE* ε4 variant being the largest known genetic risk factor for late-onset sporadic AD in a variety of ethnic groups^2^. Modifiable risk factors include smoking, physical activity, education, social engagement, cognitive stimulation, and diet^3^. Since most of the AD cases are sporadic, these lifestyle risk factors and/or co-morbid conditions are thought to have major effects on disease pathogenesis. However, the mechanisms by which these factors may condition risk to the disease remain almost elusive.

Several epidemiological studies have shown that atherosclerotic risk factors, including diabetes mellitus^4^, hypertension, and dyslipidemia^5^ increase the risk of AD in association with chronic cerebral hypoperfusion^6^. Indeed, AD patients show significantly worse cognition when they present chronic microvascular ischemic lesions, such as white matter hyperintensities^7,8^. One may hypothesize these changes derive from comorbid white matter dysfunction, which may affect brain function by interfering with inter-regional communication. However, a recent study showed that the amount of white matter alteration is associated with higher brain amyloid burden^9^, even in individuals with preserved cognition. Altogether, these data suggest that chronic cerebral hypoperfusion not only impairs the function of white matter, but also accelerates the Aβ accumulation in the human AD brain.

Studies in animal models of chronic cerebral hypoperfusion, such as the bilateral common carotid artery stenosis (BCAS) model^10^, converge with clinical studies in humans to show that chronic cerebral hypoperfusion accelerates AD pathology, including Aβ accumulation^11–13^, subsequent tau phosphorylation^14,15^, and eventual neuronal loss^12^.

Once Aβ peptide is produced through the proteolytic processing of the amyloid precursor protein (APP) by the β- and γ-secretases in the brain, it is then partly decomposed by several peptidases or cleared via the venous drainage^14,16^. Subsequent to its production, Aβ starts to assembly and form small low-molecular-weight oligomers consisting of a small number of molecules (early stage). These small oligomers engulf other Aβ monomers or small Aβ oligomers and grow into larger high-molecular-weight Aβ oligomers. At the end, this process may culminate in the formation of insoluble Aβ fibrils. However, at the same time, these oligomers start to grow and increase in molecular weight, one fraction of the fibrils or large oligomers dissociate into small oligomers or monomers. Aβ species are, thus, in a continuous and dynamic association-dissociation equilibrium^17–20^. It is a possible scenario that aberrant accumulation of Aβ under chronic hypoperfusion may derive from an imbalance in this equilibrium. Nevertheless, little is known about the mechanisms by which chronic hypoperfusion accelerates Aβ accumulation.

In this study, we induced chronic cerebral hypoperfusion in a mice model of AD to study how chronic cerebral hypoperfusion may affect the Aβ association – dissociation equilibrium during this disease. We hypothesized chronic cerebral hypoperfusion may change biochemical properties of Aβ oligomers in association with reduced dynamics of interstitial fluid in the brain parenchyma.

## Results

### Chronic cerebral hypoperfusion enlarged Aβ plaques

**Figure 1:**
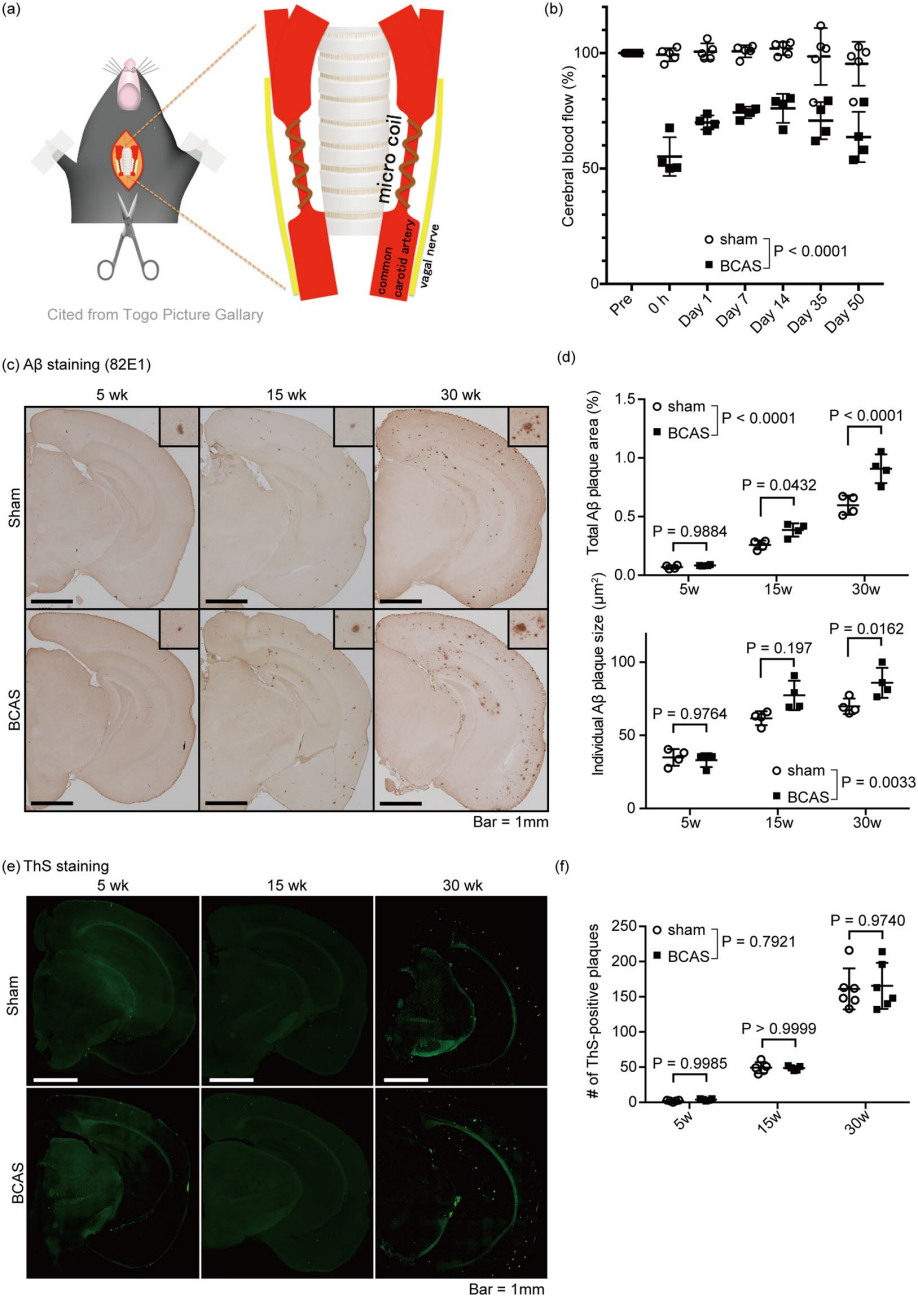
BCAS accelerated Aβ plaque growth in APP/PS1 mice. (**a**) Schema of the BCAS method. Microcoil was introduced into the common carotid artery, avoiding the vagal nerve. (**b**) Temporal profiles of cerebral blood flow in sham- and BCAS-operated mice. (**c**,**e**) Immunohistochemical detection of Aβ in the brain of sham- and BCAS-operated mice. Mice were sacrificed at week 5, 15 or 30 after the surgery. Representative immunohistochemical images are shown from a total of n = 4 mice. Insets are magnified view of Aβ plaques. Bar = 1 mm. (**d**) Quantification of panels (**c**). Total Aβ plaque area and individual Aβ plaque area are measured. Mean ± SD. n = 4 per group. (**e**) Fluorescent images of Thioflavin-S staining visualization of the Aβ-plaque core. Representative immunohistochemical images are shown from a total of n = 5 (week 5 and 15) or 6 (week 30) mice. (**f**) Quantification of panels (**e**), showing the number of Aβ plaques. Mean ± SD. n = 5 (week 5 and 15) or 6 (week 30) per group. Statistical significance was determined using Two-way ANOVA followed by *post hoc* Sidak method.

To analyze the effect of the chronic cerebral hypoperfusion on AD pathology, we applied BCAS to APP/PS1 mice harboring *APP* transgene with Swedish mutation and *PSEN1dE9* transgene^21,22^ (Fig. 1(a)). As expected, BCAS decreased cerebral blood flow (CBF) 70.0 ± 3.04% (mean ± SD) 1 day after the surgery and lasted up to 50 post-operative days (Fig. 1(b)). Replicating previous work from our research group, we found that decreased CBF induced refraction in the white matter in the cingulum, as shown by Klüver-Barrera staining, without apparent neuronal apoptosis (Supplemental Fig. S1(a,b)). BCAS had no effect on the number or the individual and total area of Aβ plaques at 5-weeks post-surgery (Mean ± SEM; Individual Area: 34.95 ± 2.84 μm^2^ in sham vs. 33.05 ± 2.36 μm^2^ in BCAS, Total Area: 0.070 ± 0.0079% in sham vs. 0.084 ± 0.0023% in BCAS, Number: 2.0 ± 0.55 in sham vs. 3.8 ± 0.58 in BCAS) (Fig. 1(c–f)). Although the number of Aβ plaques did not show any difference between sham- and BCAS-operated mice 15 and 30 weeks after the surgery, (Mean ± SEM; 49.20 ± 3.50%, 161.2 ± 11.9% in sham vs. 48.60 ± 1.36%, 165.5 ± 13.4% in BCAS, in 15 weeks and 30 weeks, respectively) (Fig. 1(e,f)), the individual and total area of Aβ plaques significantly increased in the BCAS mice (Mean ± SEM; Individual area: 61.68 ± 2.37 μm^2^, 69.82 ± 2.66 μm^2^ in sham vs. 77.31 ± 5.04 μm^2^, 85.90 ± 5.10 μm^2^ in BCAS, Total area: 0.26 ± 0.02%, 0.60 ± 0.041% in sham vs. 0.39 ± 0.028%, 0.91 ± 0.061 in BCAS, in 15 weeks and 30 weeks, respectively) (Fig. 1(c,d)), suggesting that chronic cerebral hypoperfusion accelerated the Aβ plaque growth.

To exclude the possible effects of BCAS on the production or enzymatic degradation of Aβ to increase the amount of Aβ in the brain, we first analyzed the level of proteins known to be related to Aβ production or degradation. As shown in Supplemental Fig. S2, there were no significant changes in the amounts of APP, sAPPα, sAPPβ, ADAM10, BACE1, PSEN1, PEN2, APH1, Nicastrin, Neprilysin, RAGE, LRP1, Clusterin, and ABCB1^23,24^. We then analyzed the level of Aβ in each fraction of brain homogenates. As expected, serial detergent fractionation did not reveal any significant increase of Aβ in any of fractions analyzed (Supplemental Fig. S3(a–d)). We then hypothesized that instead of having an effect on production or metabolism, chronic cerebral hypoperfusion could enhance Aβ convergence to the aggregation seed without changing the seed formation process itself. This would increase the amount of aggregation-prone Aβ species, leading to eventual enlargement of the Aβ plaque areas.

### Chronic cerebral hypoperfusion favored the formation of HMW Aβ oligomers

**Figure 2:**
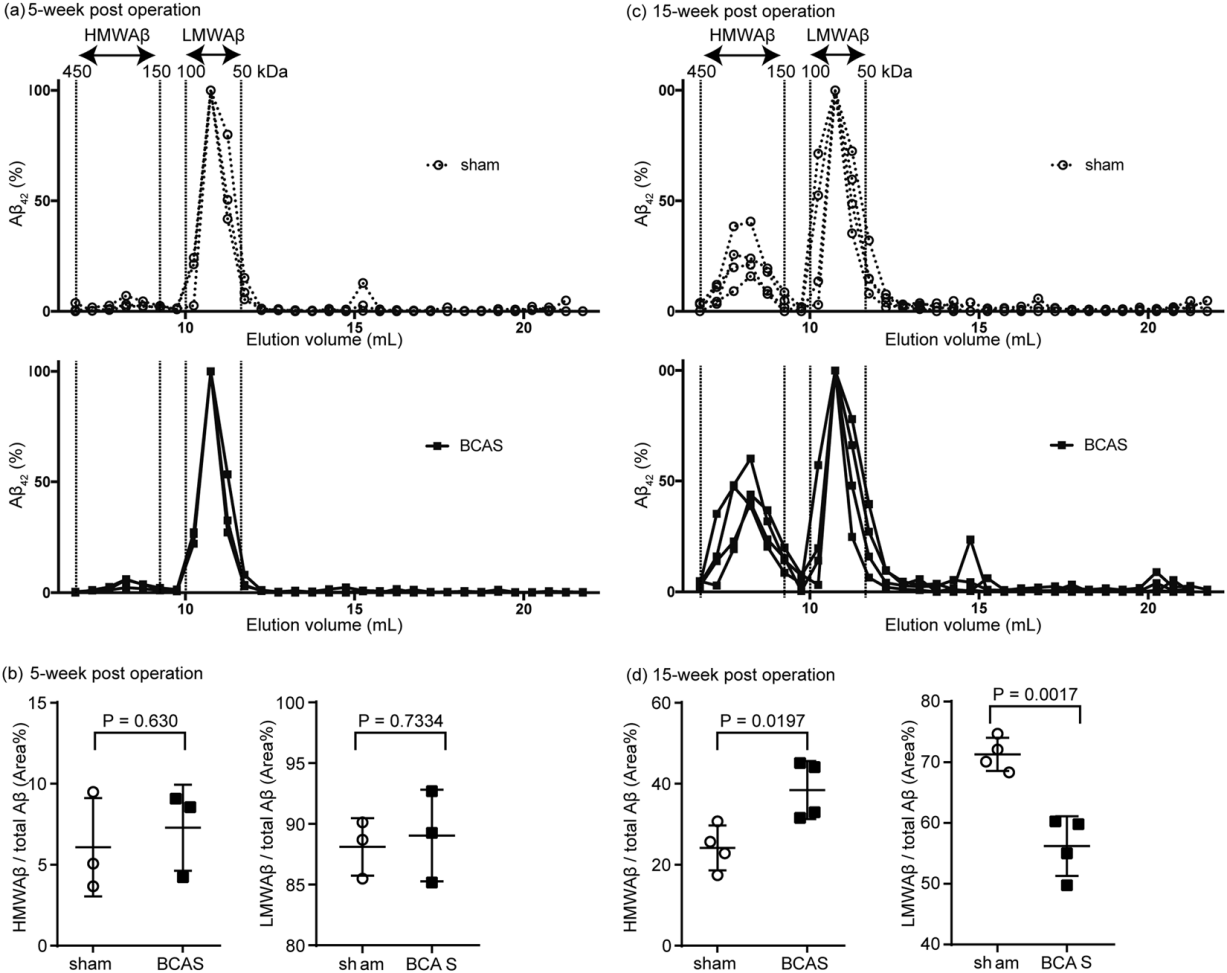
BCAS increased Aβ oligomers with high molecular weight. (**a**,**c**) Size exclusion chromatography of PBS-soluble Aβ_42_ from brain homogenate at week 5 and 15 post-operation. The concentration of Aβ_42_ was measured by ELISA, and normalized to peak-concentration of low molecular weight Aβ = 100%. The molecular size of each fraction was determined by comparison with a molecular size marker. (**b**,**d**) The percentage of high and low molecular weight species in the total amount of Aβ_42_ was determined as the area under the curve. Comparisons between groups were performed by using Student *t*-test. n = 4 per group.

To test our hypothesis, we performed size exclusion chromatography (SEC) of the PBS-soluble fraction of brain lysates from BCAS treated APP/PS1 mice to separate Aβ oligomers according to its molecular size. By modifying the previous protocol^25^, we separated Aβ oligomers roughly into two fractions: (1) a high molecular size fraction (150–450 kDa) and (2) a low molecular size (50–100 kDa) (Fig. 2(a,c)). Cerebral hypoperfusion over 15 weeks induced a relative increase of Aβ oligomers with high molecular size (Mean ± SEM; 24.2 ± 2.78% in sham vs. 38.5 ± 3.58 in BCAS) and a relative decrease of Aβ oligomers with low molecular size (Mean ± SEM; 71.32 ± 2.73% in sham vs. 56.21 ± 4.919 in BCAS), which was not observed at week 5 (Mean ± SEM; HMWAβ, 6.08 ± 1.76% in sham vs. 7.29 ± 1.53 in BCAS, LMWAβ, 88.10 ± 2.67% in sham vs. 89.04 ± 3.76 in BCAS) (Fig. 2(a–d)). Considering that the total amount of PBS-soluble Aβ at 5 weeks and 15 weeks after the BCAS surgery did not change significantly (Supplemental Fig. S3(a–d)), we concluded that Aβ oligomers with lower molecular size were assembled and shifted to species with higher molecular size.

**Figure 3:**
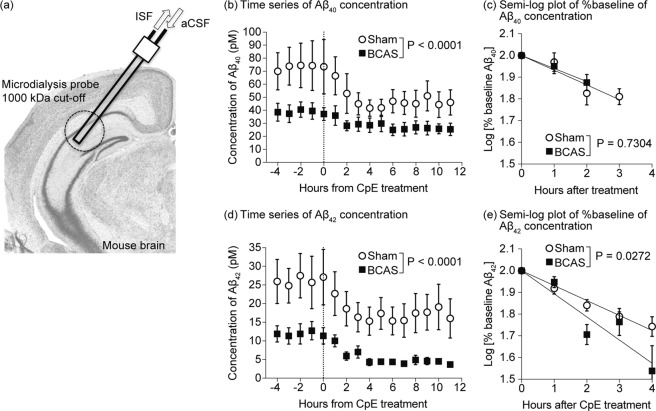
BCAS decreased the concentrations of small molecular weight Aβ, and accelerated their apparent clearance in APP/PS1 mice. (**a**) Schematic view of the *in vivo* microdialysis apparatus. The guide cannula was stereotactically implanted into the hippocampus. The microdialysis probe, equipped with 1000 kDa semipermeable membrane, was inserted into the target region through the guide cannula. Artificial cerebrospinal fluid (aCSF) was circulated within the probe every hour and collected as interstitial fluid (ISF). Aβ concentration was measured by ELISA. (**b**,**d**) Time-series of the Aβ_40_ and Aβ_42_ concentration in each fraction. A gamma-secretase inhibitor, Compound E, was administered at the time point 0 to inhibit the local production of Aβ. (**c**,**e**) Half-life periods of Aβ_40_ (**c**) and Aβ_42_ (**e**) within the hippocampus of sham or BCAS-operated mice. The concentrations from 0 h to 4 h in panels b and d were replotted and normalized with baseline concentration. Half-life periods of Aβ_1–40_ and Aβ_1–42_ were determined as the slopes of the concentrations. Statistical significance was determined by two-way ANOVA.

To directly assess if BCAS could decrease the amounts of low molecular weight Aβ oligomers *in vivo*, we analyzed the dynamics of the low molecular weight oligomeric Aβ by microdialysis (Fig. 3(a)). In this assay, a semi-permeable membrane with 1000 kDa pores allowing oligomeric Aβ molecules under 160 kDa to pass the membrane was used to enable us to measure the concentration of oligomeric Aβ in the interstitial fluid (ISF)^25–27^. By adding a γ-secretase inhibitor directly to the ISF, thus halting the production of Aβ, we evaluated the Aβ dynamics in the ISF. As expected from size exclusion chromatography, BCAS decreased the amount of Aβ in the ISF at baseline (before adding the γ-secretase inhibitor) (Fig. 3(b,d)), confirming the decreased amount of Aβ oligomers with lower molecular size. Interestingly, the apparent clearance of Aβ_42_ was accelerated in the BCAS-operated mice (t_1/2_; 4.38 hours(h) in sham vs. 2.83 h in BCAS (95% confidence interval 3.92–4.78 vs.2.15–2.83, respectively)) (Fig. 3(e)), while there was no change in the dynamics of Aβ_40_ (t_1/2_; 4.44 h in sham vs. 5.02 in BCAS (95% confidence interval 3.17–7.43 vs. 4.00–6.75, respectively)) (Fig. 3(c)), implying that Aβ_42_ could converge to the aggregation-seed faster than the Aβ_40_. Altogether these data suggest that BCAS-induced cerebral hypoperfusion shifted the Aβ equilibrium to favor the formation of assembled forms of high molecular weight.

### BCAS-induced cerebral hypoperfusion attenuated ISF dynamics

**Figure 4:**
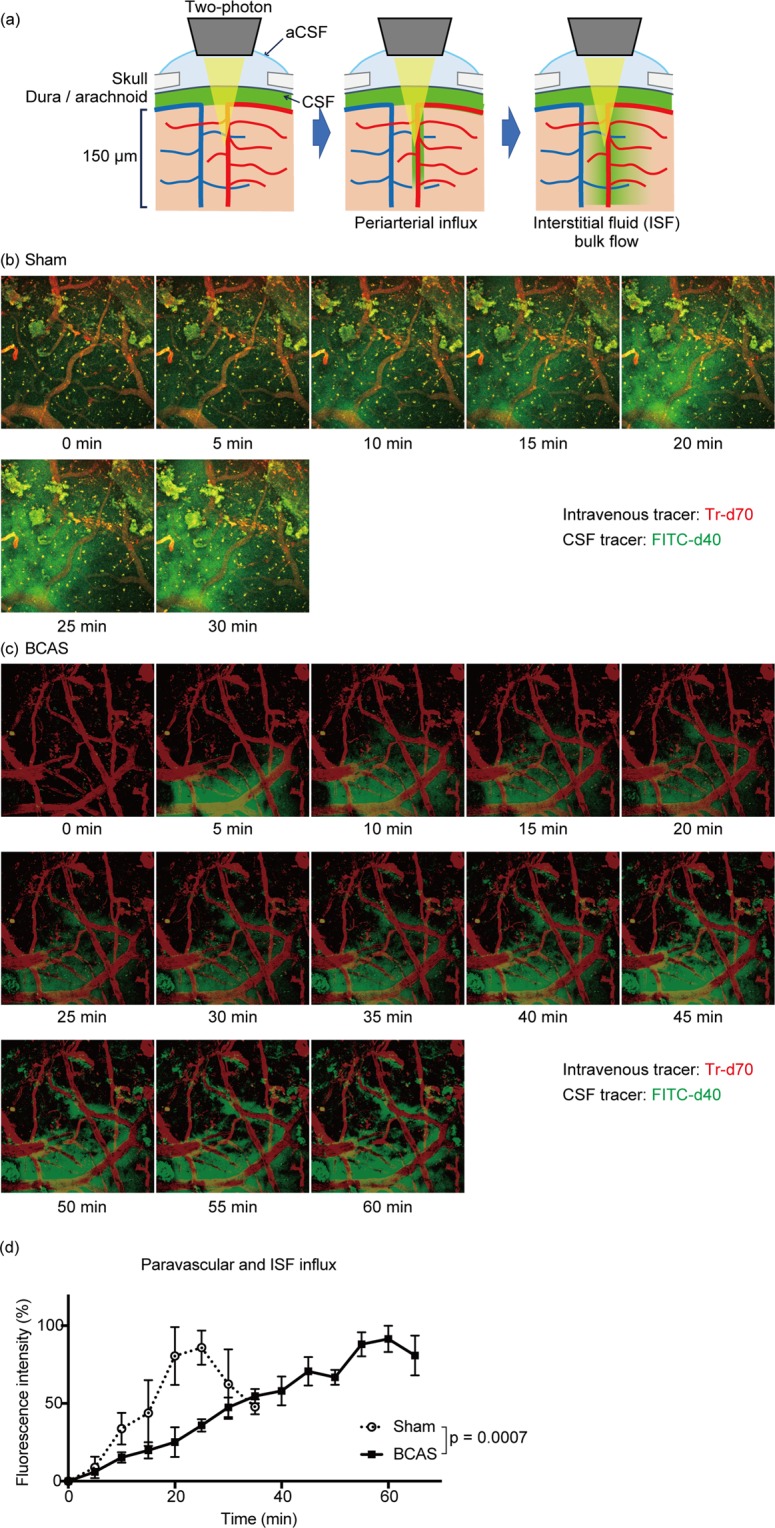
BCAS decreased the dynamics of CSF. (**a**) Schematic view of the *in vivo* two-photon microscopy apparatus. CSF was visualized with FITC-d40 injected into the cisterna magna. CSF enters the brain through the para-arterial space and spread into interstitial space. Blood vessels were visualized with Tr-d70 injected into the caudal vein. (**b**,**c**) Time series of two-photon microscopy depicting the CSF entering into the brain parenchyma in both sham- and BCAS-operated mice. (**d**) Quantification of panel b and c. Maximal fluorescence intensity was normalized to 100% in each group. Statistical significance was determined by two-way ANOVA. n = 3 per group.

We then wondered what mechanisms could be underlying these changes in the biochemical characteristics of Aβ oligomers after chronic cerebral hypoperfusion. In the human brain, atherosclerotic risk factors like hypertension and diabetes mellitus induce fibrohyalinosis in medullary arteries, which leads to hypoperfusion of the brain tissue^28^. Sclerosis of medullary arteries also decreases the arterial wall dynamics, leading to decreased influx of cerebrospinal fluid into the brain parenchyma through the para-arterial influx route^29^. Bearing this in mind, we hypothesized that the changes in the aggregation properties of the Aβ oligomers in the BCAS model could be attributed to an increase in the congestion of the interstitial fluid, due to decreased influx of cerebrospinal fluid (CSF) into the brain parenchyma. To confirm this hypothesis, we injected fluorescent tracer FITC-d40 into the cisterna magna and examined how the CSF would enter and spread from the para-arterial space into the brain parenchyma (Fig. 4(a)). As expected, BCAS decreased the speed of the flux of CSF to the para-vascular space and brain parenchyma, which supported our hypothesis of congestion of the interstitial fluid in the brain after chronic hypoperfusion (Fig. 4(b–d)).

## Discussion

**Figure 5:**
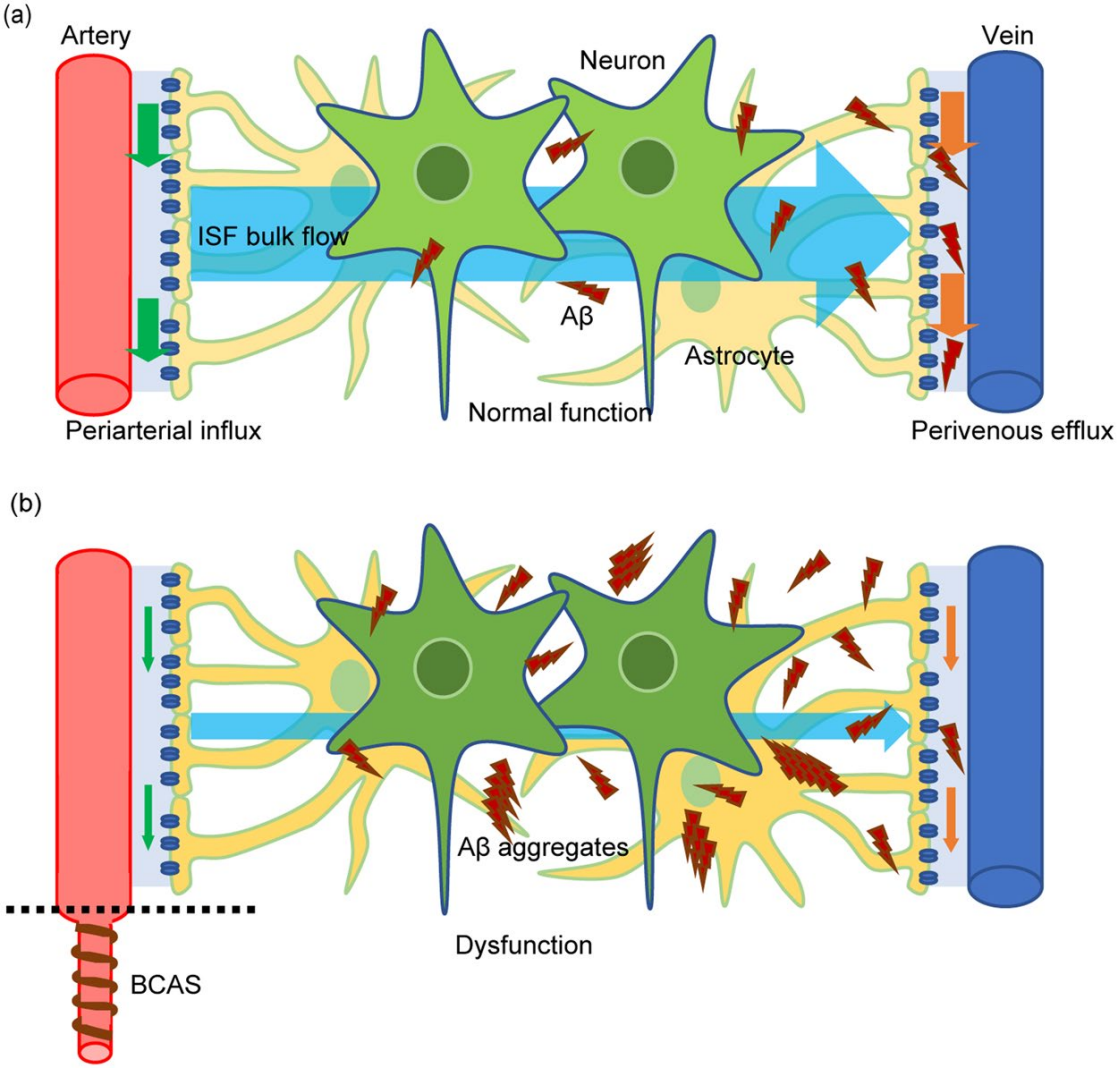
Schematic illustration summarizing our current hypothesis on how chronic ischemic condition exacerbates Aβ aggregation. (**a**) In a normal condition, periarterial influx driven by arterial blood flow enters interstitial space to facilitates ISF bulk flow, which is cleared by perivenous efflux. Dynamic ISF bulk flow keeps the association-dissociation equilibrium of Aβ oligomers, which prevents Aβ oligomers from aggregating and form Aβ plaques. (**b**) When arterial blood flow is reduced due to chronic cerebral hypoperfusion, this impairs the dynamics of the periarterial influx and the ISF bulk flow. The congestion of the ISF shifts the equilibrium of the Aβ oligomers to aggregation-prone Aβ oligomers with high molecular weight, which facilitates Aβ convergence, accelerating the growth of plaques already installed.

In this study, we applied bilateral carotid artery stenosis (BCAS) to the APP/PS1 mice model of AD to evaluate how the equilibrium of amyloid β oligomers respond to chronic hypoperfusion. We found that BCAS accelerated amyloid β (Aβ) convergence to the aggregation seed, facilitating the growth of Aβ high molecular weight species, without changing the total Aβ amount in the brain in BACS-AD mice. We suggest that cerebral hypoperfusion after BCAS shifted Aβ species equilibrium to a state where a greater number of Aβ molecules participate in Aβ assemblies to form aggregation-prone Aβ oligomers with high molecular weight. The reduced blood flow in the cerebral arteries due to BCAS attenuated the dynamics of the interstitial fluid leading to congestion, which may have facilitated Aβ aggregation. Altogether, our data suggest that cerebral hypoperfusion may accelerate AD by enhancing the tendency of Aβ to become aggregation-prone, perhaps through decreasing interstitial fluid dynamics (Fig. 5).

Among the previously published models of chronic cerebral hypoperfusion, the BACS model has several advantages; (1) higher reproducibility than the gradual stenosis model^30^, (2) chronic white matter alterations without neuronal death^10^ and (3) direct and easy application in other models, such as the transgenic AD mice here used. While chronic cerebral hypoperfusion in the human brain progresses gradually, the BCAS model used in this study reduces cerebral perfusion immediately after the operation, which may limit direct comparisons with humans (Fig. 1(b)). However, the neuropathological hallmarks observed in the BCAS mice are comparable to the chronic ischemic alterations observed in the human brain, with refraction in the white matter and no significant neuronal death in the gray matter (Supplemental Fig. S1). Thus, we consider that our hypoperfusion model presents acceptable external validity for analyzing the effect of chronic cerebral hypoperfusion on Aβ metabolism during AD.

Previous studies have shown that cerebral hypoperfusion increase the amount of Aβ plaques and of the insoluble fraction of Aβ^11^. However, the deposition of insoluble Aβ is the final stage of Aβ accumulation, and the process by which this stage may be reached remains almost elusive^6,8^. In that respect, our study focused on the soluble fraction of Aβ oligomers and discriminated between Aβ species with low and high molecular weights, by using microdialysis and size exclusion chromatography. BCAS-induced cerebral hypoperfusion increased the amount of soluble Aβ species with high molecular weight, suggesting that, while soluble Aβ is in the association-dissociation equilibrium, hypoperfusion may have deviated this equilibrium to increase the formation of aggregation-prone Aβ species with high molecular weight, even before the deposition of insoluble Aβ plaques.

In contrast to the increased amount of Aβ plaques observed in the BCAS-operated mice, the baseline concentration of Aβ was lower in the BCAS-operated when compared to sham-operated mice. Because the semi-permeable membrane used in the microdialysis assay has 1000 kDa pores allowing oligomeric Aβ molecules under 160 kDa to pass, only oligomeric Aβ species under 160 kDa can be measured in the microdialysis samples. Our exclusion chromatography results showed that BCAS-induced cerebral hypoperfusion increased the amount of Aβ species with molecular weights between 150 and 450 kDa, but not those under 150 kDa. This can account for the discrepancy between the increased Aβ plaques and the reduced concentration observed in our microdialysis samples. Furthermore, since microdialysis can only measure the concentration of Aβ species under 160 kDa, in the presence of Aβ production inhibition the half-life of the Aβ concentration in microdialysates is supposed to be associated with the speed with which Aβ species under 160 kDa aggregate into species of higher molecular weight. Supporting this idea, BCAS only shortened the half-time of Aβ_42_, increasing its proneness to aggregation, but left that of Aβ_40_ unchanged.

Recent studies have demonstrated that the glymphatic system^29,31^, where perivascular and interstitial flow facilitates removal of brain wastes, has a potential association with protein aggregation during neurodegenerative diseases. Iliff *et al*. showed that fluorescent tagged-Aβ injected in the brain parenchyma is cleared along the perivascular space, suggesting the glymphatic system participates in the clearance of Aβ^31^. Our two-photon microscopy data showed that BCAS-induced hypoperfusion significantly reduced the periarterial influx leading to congestion of the interstitial fluid in the brain parenchyma. This decrease in the ISF dynamics could thus have impaired Aβ clearance through the glymphatic system. However, biochemical analysis revealed no significant increase of the total Aβ amount in the BCAS-operated mice brain, implying that Aβ clearance itself was not deteriorated significantly. Therefore, our data suggest that facilitated aggregation of Aβ is likely to result not only from a dysfunction of the glymphatic clearance system, but also from an alteration of the biochemical properties of the Aβ itself (Fig. 5).

In the human brain, fibrohyalinosis of the medullary arteries due to atherosclerosis reduce cerebral perfusion, which is associated with white matter alterations observed during magnetic resonance imaging^32^. Fibrohyalynosis is a micro-vascular condition where a homogeneous hyaline deposition in the walls of arterioles impair their elasticity, reducing the periarterial influx of CSF and eventually the bulk flow of interstitial fluid. While the micro-coil was applied to the common carotid artery during the BCAS surgery, this procedure reduced the dynamics of the periarterial influx, mimicking the impaired dynamics of the atherosclerotic medullary arteries observed in humans. This finding strengthens our belief that this model represents a valuable tool to explore how changes in perfusion may affect the dynamic of Aβ during disease.

To conclude, we provide new insights regarding the influence of chronic hypoperfusion on the Aβ association-dissociation equilibrium during AD. We suggest that alterations of the biochemical properties of the Aβ oligomers, with formation of species of higher molecular weight prone to aggregation, alongside with alterations of the interstitial fluid dynamics, are responsible for the acceleration of AD pathology observed after cerebral hypoperfusion. These findings may pave the way for the development of new therapeutic strategies targeting this mechanism that may help to delay or prevent the installation of disease.

## Materials and Methods

### Animals

All animal procedures were approved by the Animal Care and Use Committee of the University of Tokyo (P15-061), and were performed in accordance with standard guidelines for animal experiments of the University of Tokyo. APPswe/PS1dE9 (APP/PS1) mice expressing APP KM670/671NL and PSEN1dE9 were purchased from the Mutant Mouse Regional Resource Center (MMRRC; Davis, CA, USA). C57BL6/J mice were obtained from CLEA JAPAN, Inc. (Tokyo, Japan). All mice were bred in a specific pathogen-free environment, housed at no more than 4 animals per cage in a 12-hours light/dark-cycle, and had access to food and water *ad libitum*. For the size exclusion chromatography experiments, only male mice were used. For the remaining experiments, both female and male mice were used.

### Surgical procedure of bilateral common carotid artery stenosis (BCAS)

BCAS surgery was performed as previously described. Briefly, 10–11-week old mice were anesthetized with 1.5% isoflurane (Abbott Japan, Japan). Following midline skin incision, bilateral common carotid arteries were carefully isolated from the vagal nerves, and 0.18 mm diameter microcoils (Samini, Shizuoka, Japan) were introduced into the common carotid arteries bilaterally^10^. We sutured the wound, discontinued the anesthesia and waited for recovery before returning mice to their cage. No inter-/post-surgical complications were observed. Mice were randomly assigned to BCAS or sham surgery^33^.

### Cerebral blood flow assessment

Cerebral blood flow (CBF) was measured according to a previously published method^10^. Briefly, skin overlying the right skull of sham- and BCAS-operated mice was removed under anesthesia with isoflurane. Then, a plastic guide cannula (outer diameter 5 mm, inner diameter 3 mm, length 4 mm) was perpendicularly attached to the skull, 2 mm posterior and 2 mm lateral to bregma, using dental cement (Sunmedical, Shiga, Japan). CBF was measured by inserting a 3-mm probe (Omegawave, Tokyo, Japan) into the cannula. Data were analyzed using a computer-based laser blood flowmeter (OMEGAFLO-Lab, Omegawave). CBF was recorded shortly before and after the operation, and at 1, 7, 14, 35 and 50 days after surgery.

### Mouse brain section

Mice brains were carefully removed after deeply anesthetizing mice with isoflurane (Abbvie, Inc., North Chicago, IL, USA) and immediately separated into two hemispheres by mid-sagittal dissection. Right hemisphere was placed in 4% paraformaldehyde, 0.1 M phosphate-buffer (pH 7.4) for 48 h at 4 °C, and embedded in paraffin according to standard procedures afterwards. Left hemisphere was immediately stored at −80 °C for other biochemical analysis. Paraffin-embedded samples were sectioned at a thickness of 5 μm.

### Sequential detergent fractionation assay

Sequential detergent protein extraction was performed at 5 weeks after the surgery by modifying a method published previously^34^. Brain samples were homogenized in phosphate buffered saline (PBS), supplemented with protease (Roche, Basel, Switzerland) and phosphatase inhibitors cocktails (Roche). The samples were centrifuged at 20,000 × *g* for 30 min at 4 °C, to obtain the PBS-soluble fraction. The pellets were then subjected to sequential fractionation with 1% Triton X-100/PBS, 2% N-lauroylsarcosine (Sarkosyl)/PBS, and 0.1% sodium dodecyl sulfate (SDS)/PBS in a serial manner, each time saving the centrifuged supernatants as the soluble fractions (sup) for each detergent. The SDS insoluble pellet (ppt) was re-suspended in 70% formic acid (Wako Pure Chemical Industries, Osaka, Japan), and incubated at 50 °C for 30 min. After evaporation, samples that had been suspended in formic acid were re-suspended in PBS and subjected to sonication to obtain formic acid-soluble fractions.

### Western blot analysis

Samples were incubated at 60 °C with 4 × lithium dodecyl sulfate (LDS) buffer (Invitrogen) and 1% 2-mercaptoethanol for 15 min. Samples were then loaded on 4–15% Mini-PROTEAN TGX gels (Bio-Rad, Hercules, CA, USA), separated by SDS-PAGE, and transferred to a polyvinylidene difluoride (PVDF) membrane using the Trans-Blot Turbo Blotting System and the Trans-Blot Turbo Transfer Pack (Bio-Rad). Non-specific binding was blocked with EzBlock CAS (ATTO Corporation, Tokyo, Japan) for 30 min at room temperature. Membranes were then incubated with primary antibodies in Tris-buffered saline/0.1% Tween (TBST) for 2 h at room temperature, or overnight at 4 °C. The following primary antibodies were used: Anti-Amyloid Precursor Protein (Y188, Abcam Cat# ab32136, RRID:AB_2289606, 1:5000), Anti-BACE1(EPR19523, Abcam Cat# ab183612, 1:1000), Anti-Presenilin 1 (EP2000Y, Abcam Cat# ab76083, RRID:AB_1310605, 1:5000), Anti-PEN2 (Abcam Cat# ab18189, RRID:AB_444310, 1:500), Anti-APH1α (Abcam Cat# ab12104, RRID:AB_298849, 1:250), Anti-nicastrin (Abcam Cat# ab45425, RRID:AB_2149572, 1:1000), Anti-neprilysin (EPR5904, Abcam Cat# ab126593, RRID:AB_11130255, 1:1000), Anti-RAGE (Abcam Cat# ab3611, RRID:AB_303947, 1:1000), Anti-LRP1 (EPR3724, Abcam Cat# ab92544, RRID:AB_2234877, 1:10000), Anti-clusterin (EPR2911, Abcam Cat# ab92548, RRID:AB_10585132, 1:1000), Anti-MDR1/ABCB1 (C219, Novus Cat# NB600–1036, RRID:AB_10149308, 1:500), Anti-sAPPα (2B3, IBL-America Cat# JP11088, RRID:AB_1630819, 1:250), Anti-sAPPβsw (6A1, IBL-America Cat# JP10321, RRID:AB_1630822, 1:50). Membranes were washed three times with 50 mM Tris buffered saline pH 7.4 supplemented with 0.05% Tween 20 (TBS-T) for 10 min, and then incubated with secondary horseradish peroxidase-conjugated antibodies (GE Healthcare, Little Chalfont, UK), diluted 1:2000 in TBS-T, for 1 h at room temperature. Membranes were washed three times with TBS-T for 10 min and then visualized using EzWestLumi Plus (ATTO). Images were captured by LuminoGraph I (ATTO), and were quantified by ImageJ software (http://imagej.nih.gov/ij/).

### Immunohistochemistry

Mouse paraffin sections were de-paraffinized, rehydrated, and subjected to an antigen retrieval procedure using sodium citrate buffer containing 10 mM sodium citrate pH 6.0 and 0.05% Tween 20 for 20 min at 110 °C. Immunostaining procedure was performed with the VECTASTAIN Elite ABC Kit (Vector Laboratories, Burlingame, CA, USA) according to the manufacturer’s instruction. Anti-amyloid β (N) (82E1, IBL; 1:1000) was used as primary antibody. Signal was revealed using the avidin-biotin enzyme complex (Vector Laboratories) and 3,3′-Diaminobenzidine (Sigma-Aldrich, St. Louis, MO, USA). As for visualization of aggregated Aβ, rehydrated brain sections were stained with 0.02% Thioflavin S (Sigma-Aldrich) in 80% ethanol for 15 min. The area of Aβ plaques were quantified using the ImageJ software.

### TUNEL assay

TUNEL (TdT-mediated dUTP nick end labeling) assay was performed using the Apoptosis *in situ* Detection Kit (WAKO, Osaka, Japan) according to the manufacture’s instruction. For positive controls, samples were treated with DNase I.

### ELISA

The concentrations of monomeric Aβ_40_ and Aβ_42_ in the brain homogenate and serial fractions were measured using a Human/Rat amyloid-β ELISA Kit (Wako) following the manufacturer’s instructions. Immediately before performing the ELISA assay, samples were mixed with an equal volume of 1 M guanidine hydrochloride (the final concentration of guanidine hydrochloride in the sample was 500 mM) and incubated for 30 min at room temperature to dissociate oligomerized Aβ.

### *In vivo* microdialysis

*In vivo* microdialysis experiments used to assess brain interstitial fluid (ISF) Aβ levels from awake and freely moving APP/PS1 mice at the age of 3 months, 2 weeks after BCAS or sham operation, were developed with a modification of a previously described method^26^. The guide cannula was stereotactically inserted into the hippocampus using the following coordinates: anterior-posterior −2.8 mm, medial-lateral ± 0.5 mm, dorsal-ventral −1.3 mm, from the bregma.

The probes used for *in vivo* microdialysis were equipped with 1000 kDa cut-off membrane, and connected to push (KDS101, Kd Scientific, Holliston, MA, USA) and pull pumps (ERP-10, Eicom, San Diego, CA, USA). The probes were manually inserted through the guide cannula into the target region. After probe insertion, mice were placed into cages designed to allow unrestricted movement of the animal without probe assembly tangling. To measure Aβ_40_ and Aβ_42_, microdialysis probes had a constant flow rate of 1.3 μl/min. Microdialysis samples were collected hourly using a refrigerated fraction collector.

### Size exclusion chromatography

PBS-soluble fractions of brain homogenates of BCAS- or sham-operated APP/PS1 mice were injected into a single Superdex 75 10/300GL column (GE Healthcare), in order to separate soluble Aβ species according to molecular size (at a flow rate of 0.5 ml/min) by using an AKTA explorer 10 S/100 UNICORN ver. 5.0 (GE Healthcare). The concentration of Aβ_1–42_ in each fraction was measured by–specific ELISA kits.

### *In vivo* imaging with two-photon microscopy

*In vivo* two-photon laser scanning microscopy imaging was performed 2 weeks after the BCAS or sham surgery by modifying previously published methods^31^. Anesthetized BCAS- or sham-operated C57BL/6 J mice were positioned in a stereotaxic frame, and a craniotomy (1 mm in diameter) was made over the cortex, 2 mm lateral and 2 mm posterior to bregma. The dura was left intact and the craniotomy was covered with artificial CSF (aCSF). A glass capillary was inserted into the cisterna magna and 5 µL of FITC-conjugated dextran-40 (0.5% in aCSF, Thermo Fisher Scientific, Waltham, MA, USA) was injected intracisternally. The vasculature was visualized by intravenous administration of 0.1 mL Texas Red-dextran 70 (1% in PBS, Thermo Fisher Scientific, Waltham, MA, USA), which was used to identify the paravascular space.

A laser-scanning microscope system FV1000MPE2 (Olympus, Tokyo, Japan) equipped with an upright microscope (BX61WI, Olympus, Japan), a water-immersion objective lens (XLPlanN25xW; numerical aperture, 1.05), and a pulsed laser (MaiTaiHP DeepSee, Spectra Physics, Santa Clara, CA, USA) was used for *in vivo* imaging. Excitation wavelength was 890 nm, and FITC and Texas Red signals were observed at 495–540 nm and 575–630 nm, respectively. The cortex was repeatedly scanned with dual-channel (FITC and Texas Red) 512 × 512 pixels image acquisition from the surface to 150 µm below the surface, with 5 µm z-steps, at 5 min intervals for the full duration of the experiment. Region of interest was determined based on the vasculature visualized with Texas Red-dextran 70. The images were obtained until the fluorescence intensity of FITC peaked out. CSF tracer movement through the paravascular spaces were quantified using the ImageJ software. Groups were compared using a two-way analysis of variance from 0 min to 35 min. Fluorescence intensities were normalized to a maximum intensity = 100% for each experiment.

### Statistical analyses

We used GraphPad Prism 7 software (GraphPad, San Diego, CA, USA) for the statistical analyses. Unless noted, differences between groups were tested using unpaired *t*-tests. P-values are indicated in each figure, respectively. We defined P < 0.05 as statistically significant.

## Supplementary information

Supplementary Information
