# Supplementary material for: Chronic cerebral hypoperfusion shifts the equilibrium of amyloid β oligomers to aggregation-prone species with higher molecular weight
Source: Sci Rep. 2019 Feb 26;9:2827. doi: 10.1038/s41598-019-39494-7 (PMC6391466; doi:10.1038/s41598-019-39494-7)

Supplemental Information

Chronic cerebral hypoperfusion shifts the equilibrium of amyloid β oligomers to aggregation-prone species with higher molecular weight

Taro Bannai, Tatsuo Mano, Xigui Chen, Gaku Ohtomo, Ryo Ohtomo, Takeyuki Tsuchida, Kagari Koshi-Mano, Tadafumi Hashimoto, Hitoshi Okazawa, Takeshi Iwatsubo, Shoji Tsuji, Tatsushi Toda, Atsushi Iwata

List of Figures

Supplemental Figure S1. BCAS decreased the area of corpus callosum with refraction

Supplemental Figure S2. BCAS had no effect on APP processing and Aβ metabolism in APP/PS1 mice

Supplemental Figure S3. Serial detergent fractionation assay of APP/PS1 mice brain after sham- or BCAS operation.

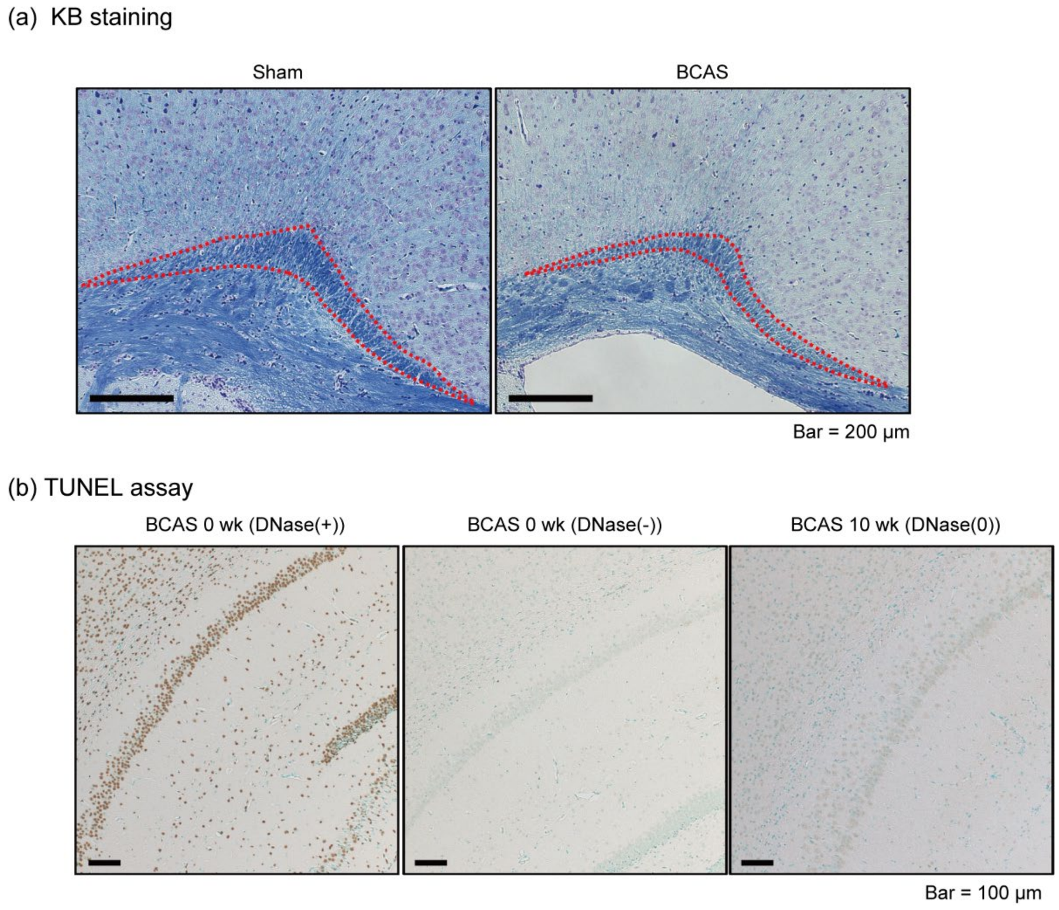

Supplemental Figure S1. BCAS decreased the area of the cingulum with refraction

(a) KB staining of the coronal section of the mice brain. The corpus callosum was surrounded by red dot-line. Bar = 200 µm. (b) TUNEL assay of the hippocampus at week 0 or 10 after BCAS operation. Assays with DNase treated sections were used as positive controls. Bar = 100 µm.

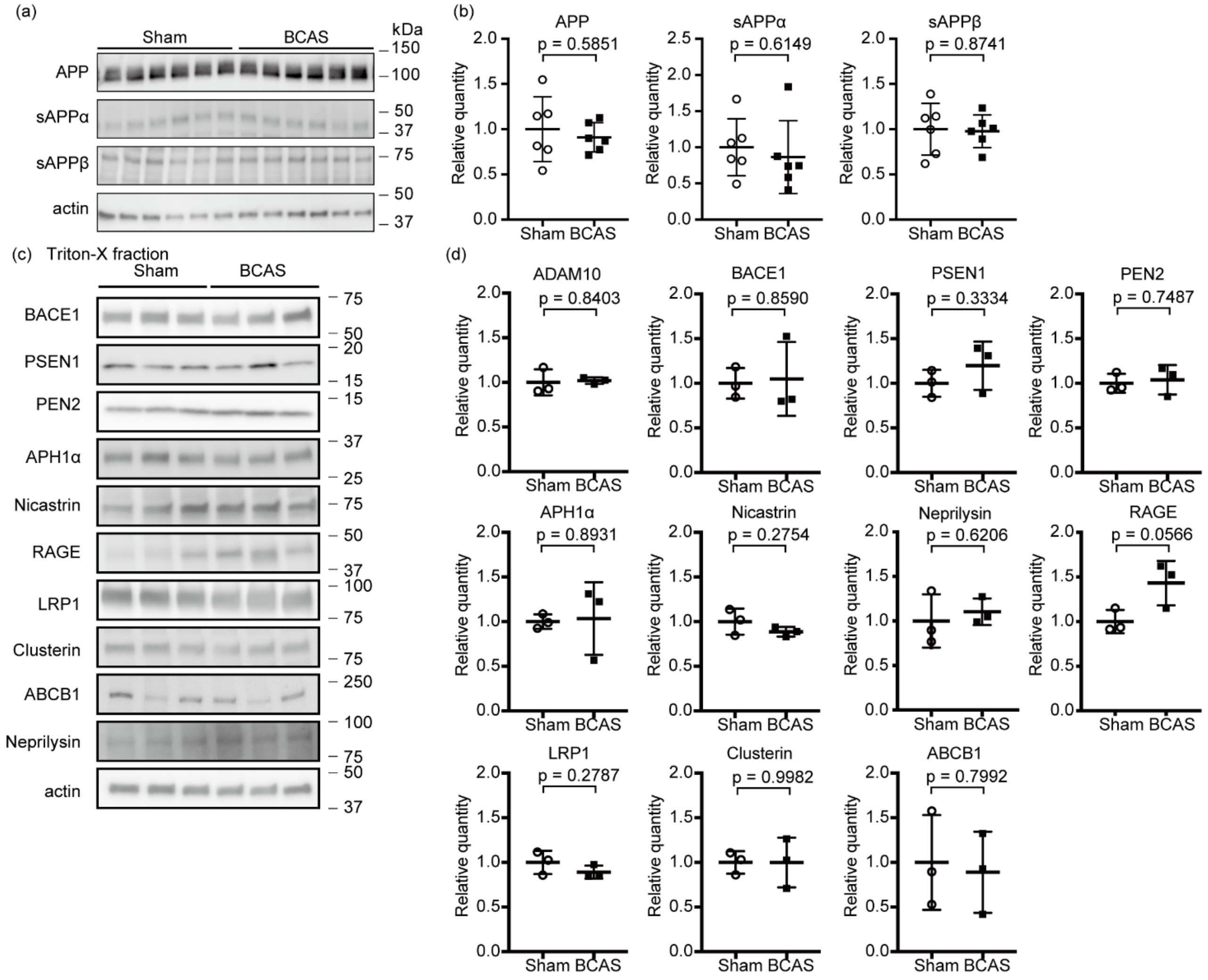

Supplemental Figure S2. BCAS had no effect on APP processing and Aβ metabolism in the brain of APP/PS1 mice

(a) Quantifications of APP, sAPPα and sAPPβ in the Triton-X fraction by western-blot. (b) Quantification of panel (a). Actin-normalized level of expression of each protein in the Triton-X fraction was normalized to sham = 1. (c) Quantifications of APP and Aβ processing enzymes in the total brain homogenates by western-blot. (d) Quantification of panel (d). Relative quantity of each protein to actin expression in Triton-X or PBS fraction was normalized to sham = 1.

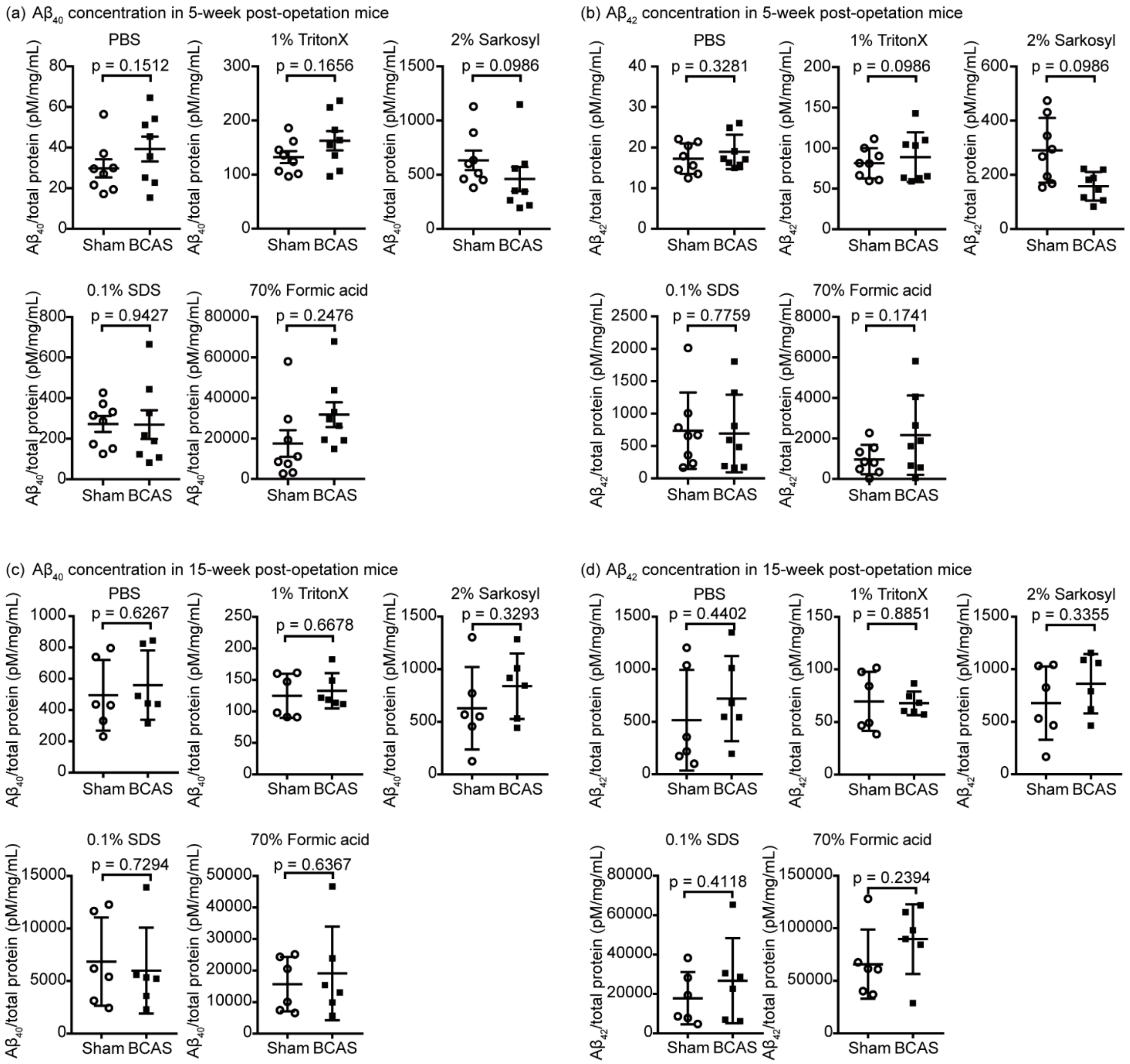

Supplemental Figure S3. Serial detergent fractionation assay of sham- or BCAS-operated APP/PS1 mice brain homogenates.

The concentration of $A\beta_{40}$ (a, c) and $A\beta_{42}$ (b, d) in the brain homogenates was measured by ELISA. Samples were obtained at 5 weeks (a, b) and at 15 weeks (c, d) after the BCAS operation. Statistical significance was determined by using a Student's *t*-test. n = 8 (5 weeks) and 6 (15 weeks).

Supplemental Figure S2A

APP

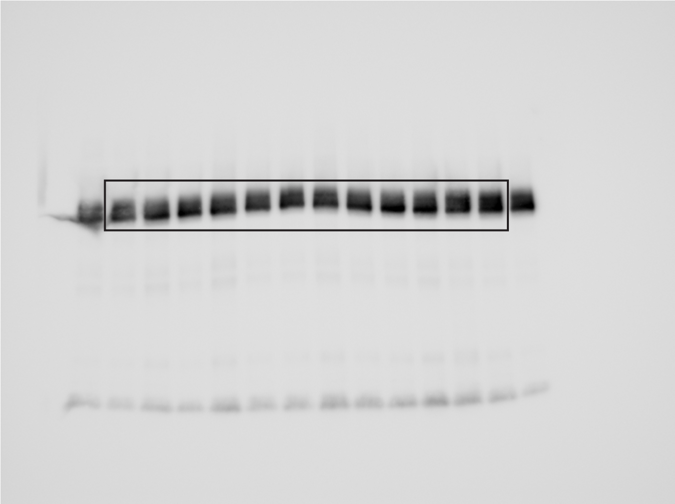

sAPPα

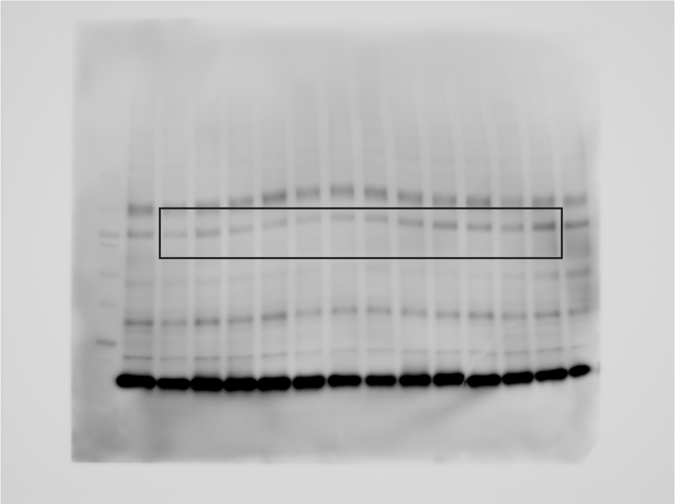

sAPPβ

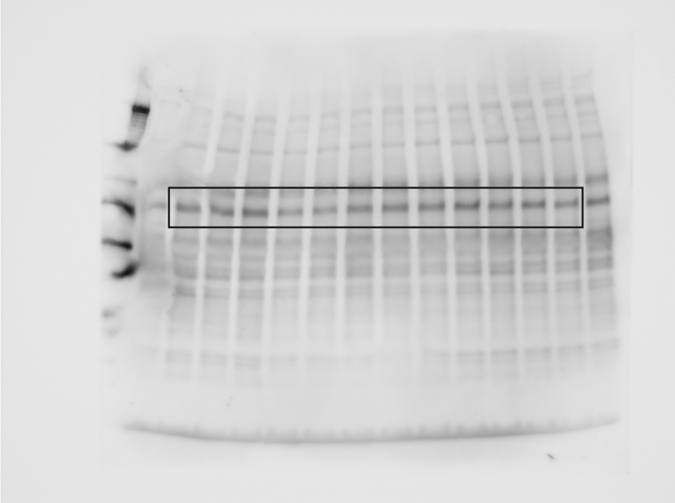

Actin

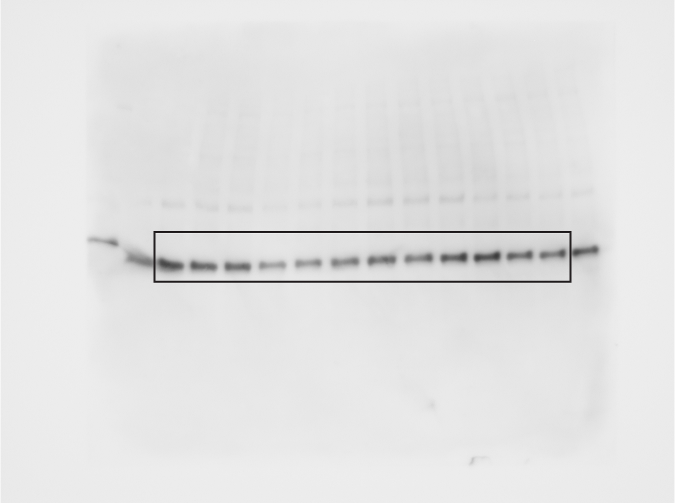

Supplemental Figure S2C

BACE1

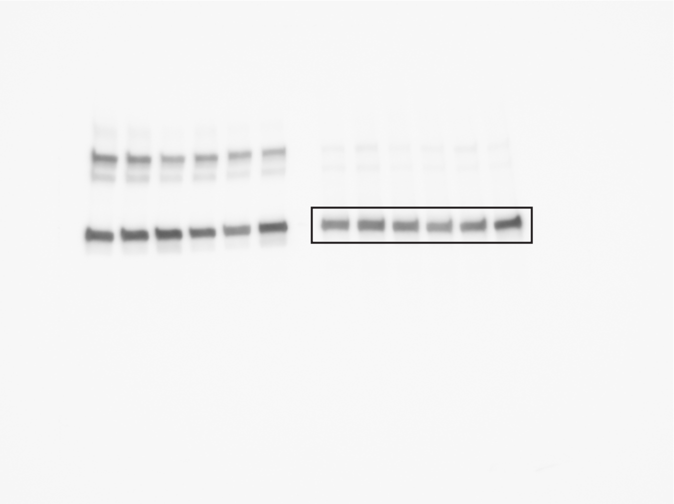

PSEN1

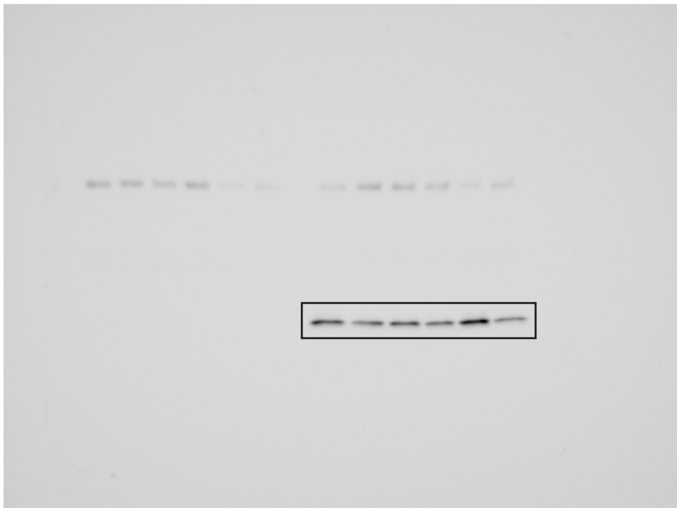

PEN2

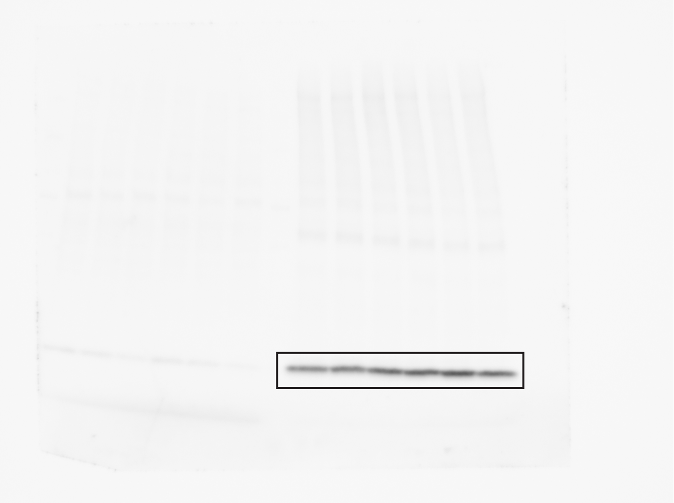

APH1α

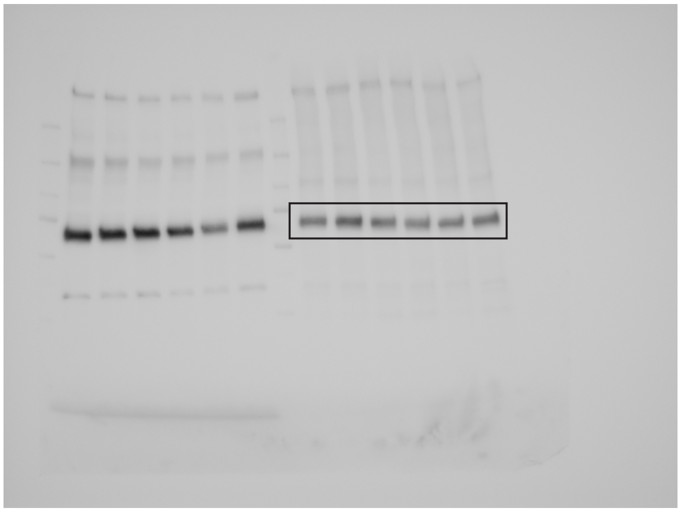

Nicastrin

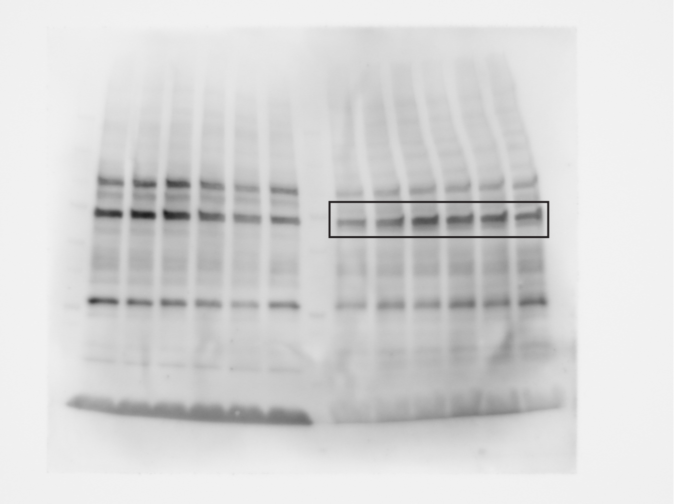

RAGE

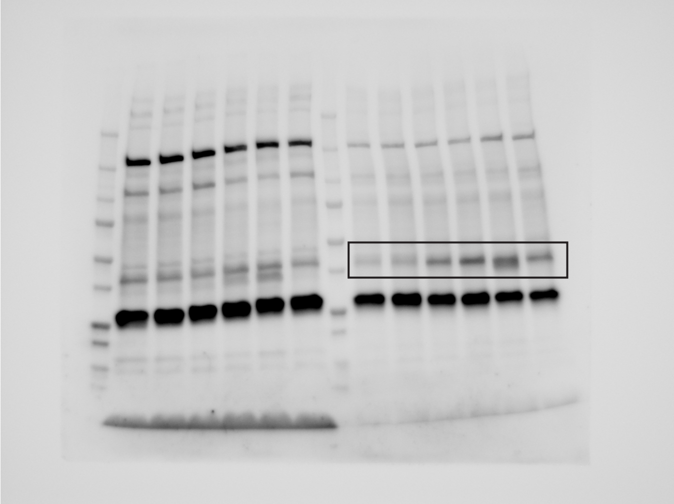

Supplemental Figure S2C

LRP1

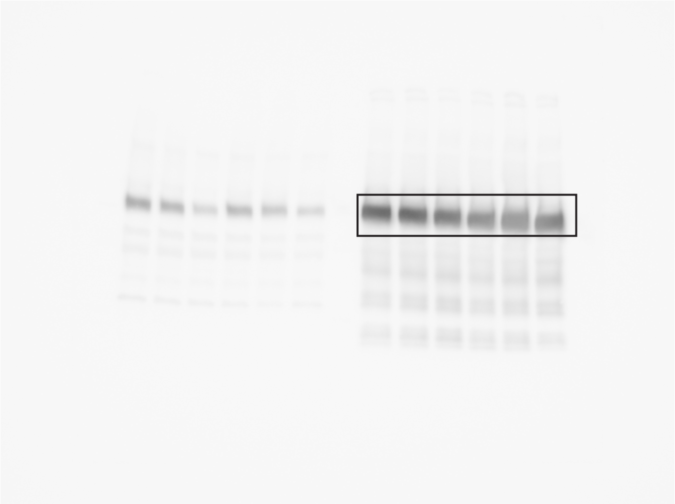

Clusterin

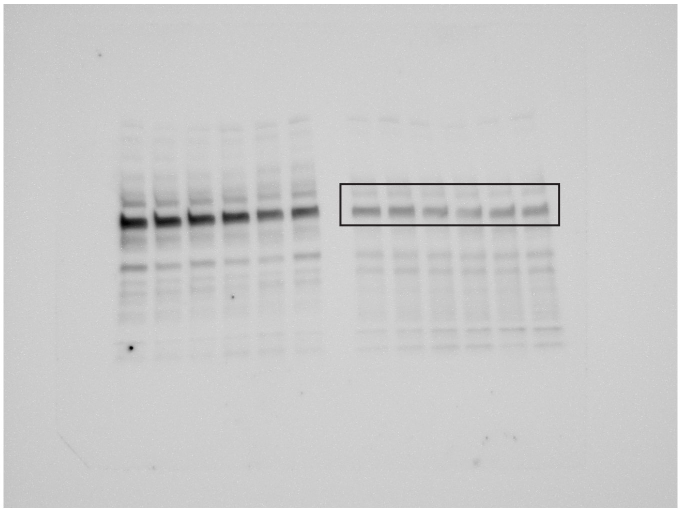

ABCB1

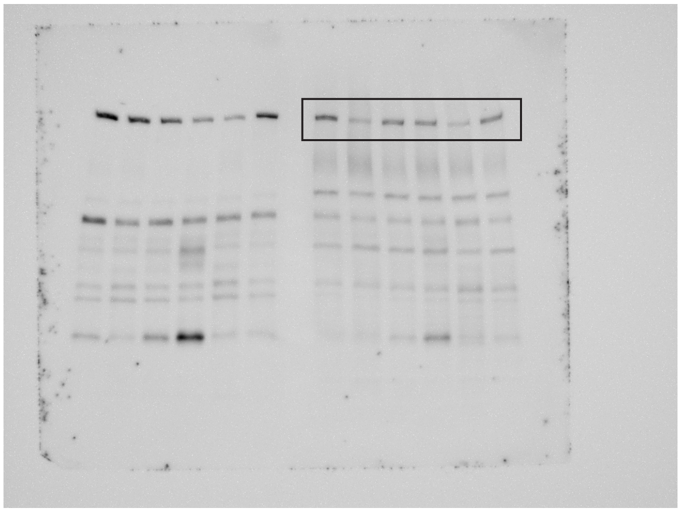

Neprilysin

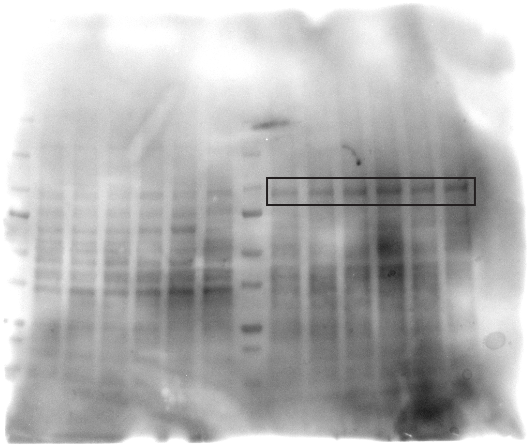

Actin

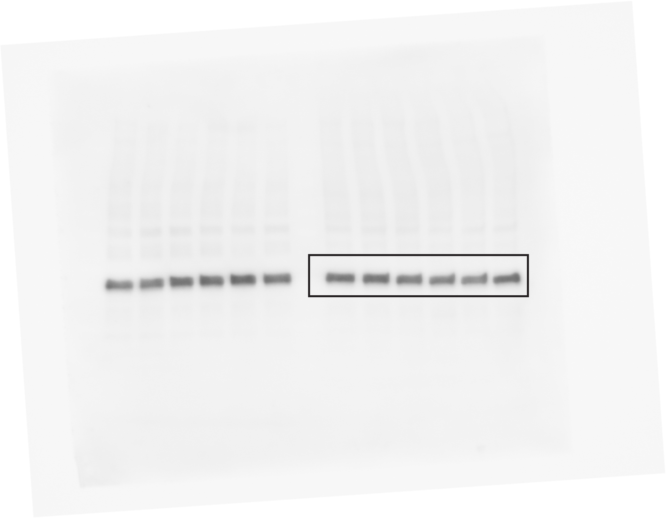

Supplement: Supplementary file 1 — Supplementary Information [file 41598_2019_39494_MOESM1_ESM.pdf]
